# RCARE: RNA Sequence Comparison and Annotation for RNA Editing

**DOI:** 10.1186/1755-8794-8-S2-S8

**Published:** 2015-05-29

**Authors:** Soo Youn Lee, Je-Gun Joung, Chan Hee Park, Ji Hye Park, Ju Han Kim

**Affiliations:** 1 Seoul National University Biomedical Informatics (SNUBI) and Systems Biomedical Informatics Research Center, Division of Biomedical Informatics, Seoul National University College of Medicine, Seoul 110799, Korea

**Keywords:** RNA editing, RNA-DNA difference, RNA-seq;software

## Abstract

The post-transcriptional sequence modification of transcripts through RNA editing is an important mechanism for regulating protein function and is associated with human disease phenotypes. The identification of RNA editing or RNA-DNA difference (RDD) sites is a fundamental step in the study of RNA editing. However, a substantial number of false-positive RDD sites have been identified recently. A major challenge in identifying RDD sites is to distinguish between the true RNA editing sites and the false positives. Furthermore, determining the location of condition-specific RDD sites and elucidating their functional roles will help toward understanding various biological phenomena that are mediated by RNA editing. The present study developed RNA-sequence comparison and annotation for RNA editing (RCARE) for searching, annotating, and visualizing RDD sites using thousands of previously known editing sites, which can be used for comparative analyses between multiple samples. RCARE also provides evidence for improving the reliability of identified RDD sites. RCARE is a web-based comparison, annotation, and visualization tool, which provides rich biological annotations and useful summary plots. The developers of previous tools that identify or annotate RNA-editing sites seldom mention the reliability of their respective tools. In order to address the issue, RCARE utilizes a number of scientific publications and databases to find specific documentations respective to a particular RNA-editing site, which generates *evidence levels *to convey the reliability of RCARE. Sequence-based alignment files can be converted into VCF files using a Python script and uploaded to the RCARE server for further analysis. RCARE is available for free at http://www.snubi.org/software/rcare/.

## Introduction

RNA editing is the post-transcriptional alteration of a single nucleotidesequence in primary messenger RNA (mRNA) or non-coding RNA (ncRNA) including micro-RNA (miRNA) transcripts [1]. The most widespread type of RNA editing in mammals is A-to-I editing, which is mediated by the adenosine deaminases acting on RNA (ADAR) enzyme [2-4]. Recent developments in DNA and RNA sequencing (RNA-Seq) technologies have led to the rapid identification of several forms of RNA editing in human cell lines, including G-to-A, C-to-U, T-to-C, C-to-A, G-to-C, T-to-A, and A-to-T [5,6]. Most of the RNA editing or RNA-DNA difference (RDD) sites are located within intron, 5' untranslated region (UTR), 3'UTR [7], and Alu sequences. However, when RNA editing events occur in the coding region, they can cause nonsynonymous protein coding substitutions, alternative splicing, and changes in gene expression [7-9]. In addition, RNA editing can affect the activity of ncRNAs such as miRNA, siRNA (short interfering RNA), and piRNA (piwi-interacting RNA) [10]. Many RNA editing events are associated with a variety of human diseases such as epilepsy, brain ischemia, depression, and brain tumors [1].

In the last few years, huge amounts of DNA and RNA-Seq data have been generated and stored in public repositories such as GEO (http://www.ncbi.nlm.nih.gov/geo/) and ENCODE (http://genome.ucsc.edu/ENCODE/). In response, many tools have been developed for detecting or annotating RNA editing and RDD sites. Prediction tools for novel RNA editing sitessuch as rddChecker (http://genomics.jhu.edu/software/rddChecker/) perform RNA-DNA sequence comparisons with RNA-Seq and genomic DNA sequence data. The rddChecker searches for RDDs, filters known single-nucleotide polymorphisms, and determines novel RNA editing sites by comparison with known RNA editing sites. However, the rddChecker is known to suffer from false-positive results. And many of the RNA editing sites reported by Li et al. [11] were subsequently demonstrated to be false positives [12,13]. From this point of view, a major challenge in identifying RNA editing or RDD sites is distinguishing between the true RNA editing sites and the false positives.

Several RNA editing-site databases have been developed that include information gathered from the literature or from manually accrued datasets such as DARNED (a database of RNA editing in humans)[14] and RADAR (a rigorously annotated database of A-to-I RNA editing)[15]. DARNED is a famous database that provides about 42,000 human RNA editing sites, while RADAR provides 1,343,464 human, 7,272 mouse, and 3,155 fly-tissue-specific RNA editing sites. Web-based RNA editing site annotation tools have been developed to take advantage of these databases. For example, ExpEdit[16] is an annotation tool that use the DARNED system for RNA editing sites. ExpEdit increases reliability by using more reliable data compared toother prediction tools, but is not able to identify novel RNA editing sites. It provides a user-friendly web interface for uploading raw RNA-Seq data such as FASTQ, SAM (sequence alignment map), and BAM (binary alignment map) files, and for exploring RNA editing. However, uploading raw RNA-Seq data cannot be completed within a practical time frame with this tool (i.e., it takes approximately 28 h to upload 700-MB BAM files). The Python package REDItools, which were developed to overcome the long uploading problem[17], perform novel RNA editing site detection and known RNA editing site annotation using DNA sequence and RNA-Seq data together from the same sample/individual or RNA-Seq data alone. VIRGO[18] is a web-based tool for identifying putative A-to-I editing sites in DNA sequences. However, Expedit and REDItools do not provide validity or reliability levelsfor each RNA editing site in the results data, and VIRGO provides only an A-to-I RNA editing form.

This study presented a new tool for RNA-Seq comparison and annotation for RNA editing (RCARE), which determines condition-dependent RNA editing sites, provides rich systematic annotations and the *evidence level*of each RNA editing site. It identifies novel RNA editing sites in the same/individual DNA sequence and RNA-Seq variant call format (VCF) data, and delivers 'executive summary' plots for the annotation and comparison of multiple samples with RNA-Seq data through a user-friendly web interface. It also provides a Python script that users can implement to preprocess raw RNA-Seq data and convert FASTQ and BAM files into RNA VCF files within a practical time frame on a desktop computer. RCARE is freely available at http://www.snubi.org/software/rcare/.

## Method

### Data collection

RCARE integrates 314,880, 6,830, and 13,018 human mRNA editing sites downloaded from DARNED (NCBI37/hg19) [14] and human ENCODE RNA-Seq data [1], and Bahn et al, respectively. [5]. The reference-based *evidence level *of each RNA editing site was generated by downloading 1,379,404 and 10,115 human RNA editing sites (hg19) from RADAR [15] and Li et al. [11]. We also downloaded Homo_sapiens.GR-Ch37.69.gtf and RepeatMasker database information from Ensembl (http://ensembl.org) and UCSC (http://genome.ucsc.edu/buildGRCCh37/hg19) to annotate the RDD site into one of the following categories: ncRNA, Ensembl Gene (ENSG) ID, Transcript (ENST) ID, Exon ID (ENSE), and repetitive element (Alu,nonrepetitive). ANNOVAR (http://www.openbioinformatics.org/annovar/) [19] was also used to annotate the following genomic features: intron, intergenic, splicing region, downstream, upstream, 3'UTR, 5'UTR, and synonymous/nonsynonymous information (Table 1).

**Table 1:** Database resources for RNA editing, listing the sources of data currently included in RCARE, specific entries in the database, and the total unique RNA editing site covered by each data type.

	No. of tissue types	No. of RNA editing sites	No. of Alu sequences	No. of references
DARNED	29	314,880	15,783	34
ENCODE	27	6,840	347	18
RADAR	30	291,901	14,318	31
Bahn et al. [5]	27	12,810	2,916	15
Li et al. [11]	1	1	0	1
Total	114	626,432	33,364	99

### RNA-Seq data preprocessing

**Figure 1:**
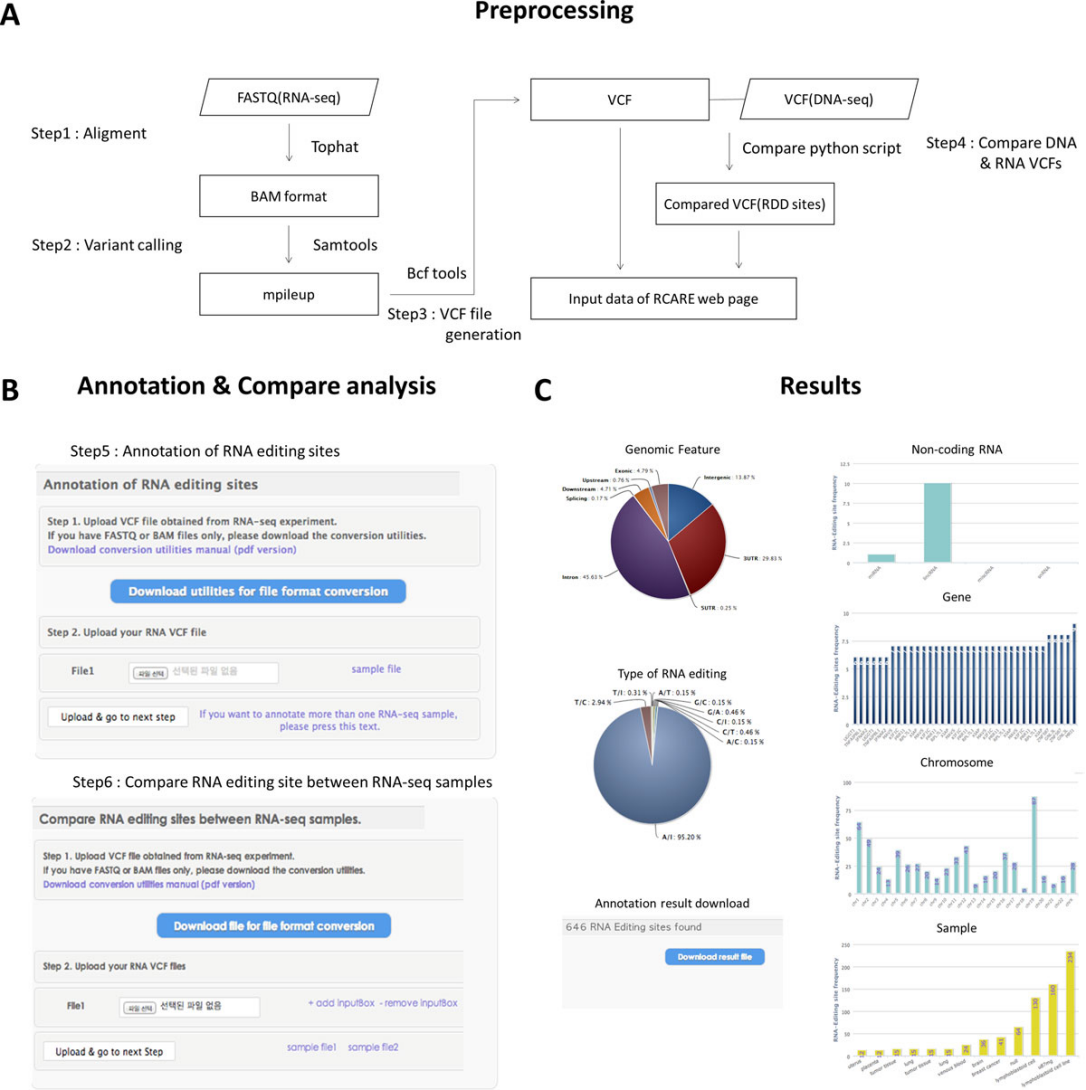
**RCARE RNA-Seq data processing steps and annotation result visualization**. A) Conversion utility RNA-Seq data processing pipeline. B) Web interface for annotation and comparison analyses. C) Summary plots of annotation results

RCARE provides a Python script for the automated conversion of a FASTQ or BAM file obtained from an RNA-Seq experiment into a VCF file with the hg19 reference genome (Figure 1A). TopHat [20], SAMtools [21], VCFtools [22], Tabix [23], and Bowtie2 [24] are run by the downloaded script on the client side to avoid the time-consuming uploading of huge RNA-Seq data. The parameters for TopHat and SAMtools are easily modifiable. Conversion utility consists of 2 types. The full version Conversion utility contains indispensable tools such as tophat, python script for BAM to VCF conversion, python script for DNA/RNA sequence variant comparison for finding novel RDD or RNA editing candidate sites and sample files for testing analysis. However, if a user already installed tools such as tophat, samtools and tabix, the user could download the light version Conversion utility. The light version excludes the aforementioned tools for faster downloads. Details of the data-processing pipeline are shown in Figure 1. If the user wants to use other tools for read mapping and variant calling, he could use other tools such as STAR and GATK, respectively, to create a VCF file as input for Annotation or *Known-RNA-editing-site-compare functions*. When both the DNA sequencing and RNA-Seq data are available, the *RCARE RDD-compare function *can be used to compare the paired VCF files created from those data. It is based on a python script and performs a comparison between RNA and DNA sequence variants according to chromosome, position, and reference/alternative sequences. This compare function can detect novel RDD or RNA editing candidate sites from DNA and RNA-VCF files. More precise detection of RNA editing sites requires both DNA and RNA VCF files obtained from the same/individual sample.

### Comparison RNA editing sites

In RCARE, *Known-RNA-editing-site-compare functions *are functions which identifies RNA sites that are differentially edited between samples according to chromosome, position, and reference/alternative sequence. It also identifies RNA editing sites intersecting between input RNA-Seq samples. A detailed method for compare functions was described in Fig S3.

### Construction of the web interface

RCARE is a web-based application that was created using HTML5 (Hypertext Markup Language 5), CSS3 (Cascading Style Sheets 3), jQuery, and Highcharts API (http://www.highcharts.com/). It also provides four functionalities: RNA-Seq data preprocessing, comparison, annotation, and visualization.

## Result

**Figure 2:**
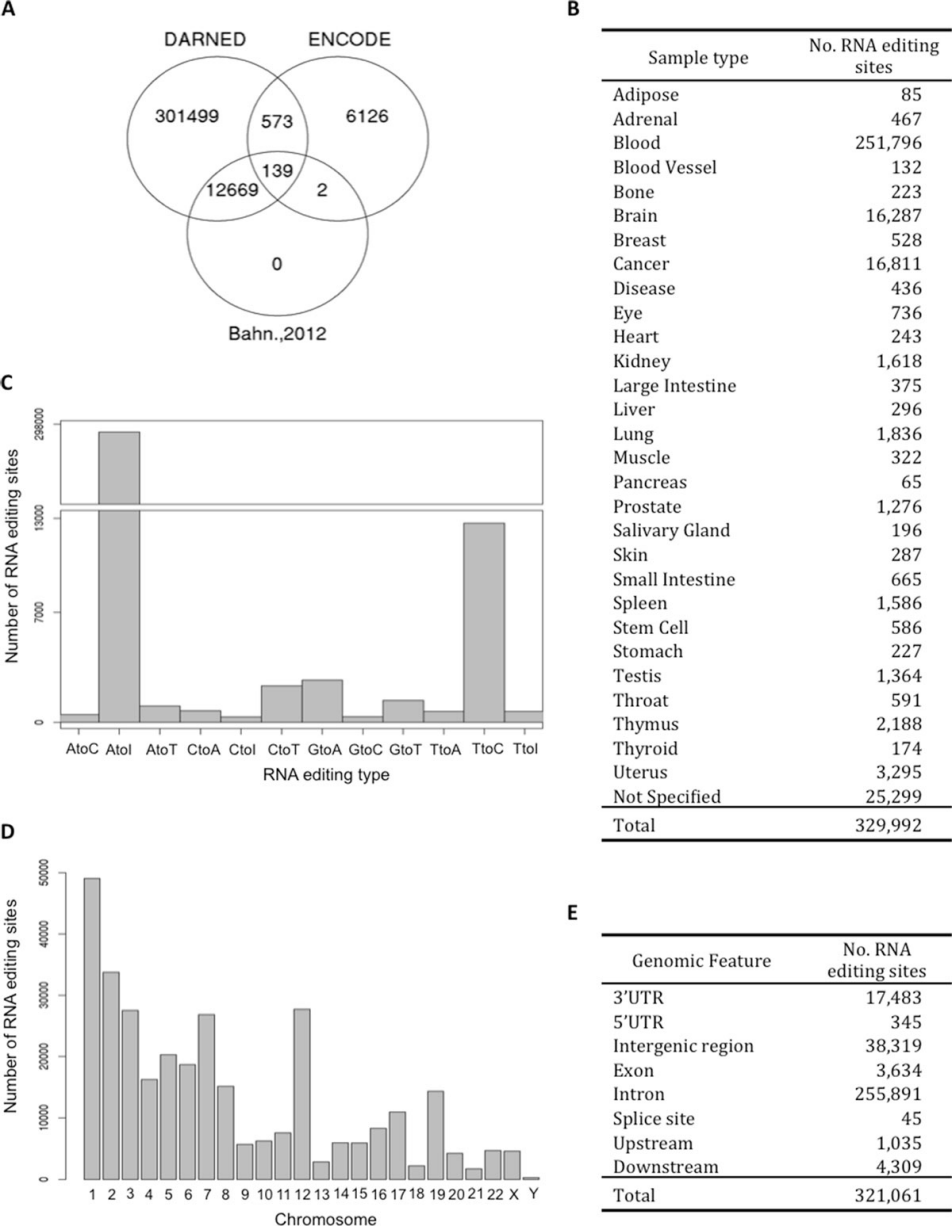
**Data compositions**. A) Venn diagram representing the number of RNA editing sites shared between DARNED, ENCODE, and Bahn et al. [5]. B) Number of RNA editing sites for each sample type. C) Bar plot representing the number of RNA editing sites for each editing type. D) Bar plot representing the number of RNA editing sites for each chromosome. E) Number of RNA editing sites for each genomic feature.

We collected 321,008 RNA editing sites from DARNED (NCBI37/hg19) [14] and human ENCODE RNA-Seq data [1], and Bahn et al. [5]. The data sets comprised 154 samples in 30 sample types, 23 papers, and 11,299 genes with 12 different types of RNA editing sites (Figure 2). RCARE annotates each RNA editing site with nine annotation categories. The RCARE annotation function checks whether an editing site: (1) creates synonymous vs. nonsynonymous changes, (2) is located at a splicing junction, (3) has certain genomic features, (4) is Alu-associated, and (5) is located in a ncRNA; it also annotates (6) gene information (i.e., gene symbol, various IDs; see additional files), (7) sample origin, (8) reference articles, and (9) the reference-based *evidence level*. In addition, it provides the unique RNA editing sites of each sample and the intersection/difference between all pairwise sample comparisons with 17 useful biological annotations (Table 2).

**Table 2:** Format of RCARE annotation Results.

Column number	Column name	Definition	References
1	Chr	Chromosome of the RNA editing site in the reference genome.	[5,11,14]
2	Pos	Coordinate of the RNA editing site in the reference genome.	
3	In DNA	Base of the RNA editing site in the DNA reference sequence.	
4	In RNA	Base of the RNA editing site in the RNA sequence of sample.	
5	Gene	Gene name to which the RNA editing site belongs.	
6	*Evidence level*	The *evidence level *consists of five levels (A-E), where A is highest level (e.g., if an RNA editing site had level "A," it appeared in all five of the resource databases/papers used). *Level A: The RNA editing site appeared in five resources (evidence No. 5). *Level B: The RNA editing site appeared in four resources (evidence No. 4). *Level C: The RNA editing site appeared in three resources (evidence No. 3). *Level D: The RNA editing site appeared in two resources (evidence No. 2). *Level E: The RNA editing site appeared in one resource (evidence No. 1).	[5,11,14,15] RepeatMasker
7	Strand	+ for positive strand; - for negative strand.	[5,11,14]
8	Source	This field contains information regarding the tissue source from which the RNA editing instance was obtained.	
9	PubMed ID	This field provides the reference article from which the RNA editing data was extracted.	
10	Alu	This field provides information of Alu at the RNA editing site.	RepeatMasker
11	Data reference	Reference database.	Each database or reference
12	ENSG	Ensembl Gene ID.	GTF(*Homo sapiens*,
13	ENST	Ensembl Transcript ID.	
14	ENSE	Ensembl Exon ID.	GRCH37.17) in Ensembl
15	Genomic feature	Genomic feature of the RNA editing site. *Exonic: the variant overlaps a coding exon. *Splicing: the variant is within 2 bp of a splicing junction. *ncRNA: the variant overlaps a transcript without coding annotation in the gene definition. *5'UTR: the variant overlaps a 5' untranslated region. *3'UTR: the variant overlaps a 3' untranslated region. *Intronic: the variant overlaps an intron. *Upstream: the variant overlaps the 1-kb region upstream of the transcription start site. *Downstream: the variant overlaps the 1-kb region downstream of the transcription end site. *Intergenic: a variant is in the intergenic region.	[19]
16	Synonymous or nonsynonymous	Synonymous or nonsynonymous substitutions at the RNA editing site.	[19]
17	Noncoding RNA	This field indicates whether the location of an RNA editing site is in ncRNA.	GTF (*Homo sapiens*, GRCH37.17) in Ensembl

Although the detection of RNA editing sites is important, the reliability of the RNA editing site is even more so. To enhance the reliability of the detected RNA editing sites, we generated *Evidence level*s according to the number of resources reporting corresponding RNA editing. First, we collected five RNA editing site-related resources (DARNED, RADAR, Bahn et al. [5], Li et al. [11], Park et al. [12]) and Alu sequence information (RepeatMasker, http://www.repeatmasker.org/). Second, we integrated aforementioned resources according to chromosome, position, and reference/alternative sequences and generated *evidence level*s according to the number of resources, which addressed corresponding RNA editing sites. *Evidence level*s consist of five levels (A-E), where A is the highest level (Table 3, Additional file 1). For example, if one RNA editing site is assigned level A, it appears in all five of the databases/papers searched. Whereas, the lowest class (E) represents the lowest level of evidence for RNA editing sites, whereby they appear in only one of the reference resources; 85% of RNA editing sites in integrated data belong to class D, appearing in two of the resources. This allows the user to decide whether a detected site is a false positive or a true positive, according to the graded scale of the *evidence level*.

**Table 3:** Evidence level classification scheme for RNA editing.

Class	**Evidence No**.	Composition of evidence	Count
A	5	DARNED, ENCODE, Bahn et al. [5], RADAR, Alu	33
B	4	DARNED, RADAR, Bahn et al. [5], Alu	2,204
		DARNED, ENCODE, RADAR, Alu	23
		DARNED, ENCODE, Bahn et al. [5], RADAR	106
C	3	DARNED, Bahn et al. [5], RADAR	7,037
		DARNED, Bahn et al. [5], Alu	679
		DARNED, ENCODE, RADAR	550
		DARNED, RADAR, Alu	12,038
		DARNED, RADAR, Li et al. [11]	1
		ENCODE, Bahn et al. [5], RADAR	1
		ENCODE, RADAR, Alu	20
D	2	DARNED, Alu	806
		DARNED, RADAR	269,547
		DARNED, Bahn et al. [5]	2,749
		ENCODE, Alu	271
		ENCODE, RADAR	341
		ENCODE, Bahn et al. [5]	1
E	1	DARNED	19,107
		ENCODE	5,494
	Total		321,008

The web interface consists of three sections: user manual, download, and analysis. The user manual section provides instructions regarding the conversion utilities, annotation, comparison, and results description (Figure 1B). All manual files can be downloaded as PDF files. The download section provides conversion utilities that can be downloaded. We provide two versions of the conversion utilities: full and light. The full version includes all RNA-Seq processing tools such as TopHat, while the light version does not. The analysis section provides annotation and omparison analysis. The annotation part provides 16 useful biological annotations and the *evidence level *of each RNA editing site from the VCF format of the RNA-Seq data. The compare part provides the RNA editing sites of each sample and the intersection/difference between all pairwise samples with 16 useful biological annotations and *evidence level*s. The results page provides summary plots based on annotation categories including the distributions of genomic features, genes, ncRNAs, synonymous vs. nonsynonymous changes, types of RNA editing, and the distributions of detected RNA editing sites in each chromosome or sample (Figure 1C). All image files for plots and annotation files can be downloaded. The test results with three RNA-Seq data sets for MCF-7 (a breast cancer cell line), HUVEC (a human umbilical vein endothelial cell line) and HeLa-S3 (a cervical carcinoma cell line) downloaded from ENCODE (http://genome.ucsc.edu/ENCODE/) yielded 2437, 646 and 1190 RNA editing sites, respectively; we obtained 205, 605 and 334 RNA editing sites that had *evidence level*s of A-C from HUVEC, MCF-7 and HeLa-S3; however, these represent only 31.7%, 24.8% and 28.07% of the total RNA editing sites. This finding shows that a good *evidence level *is essential for RNA editing detection and annotation analysis. We also accomplish the *Known-RNA-editing-site-compare functions *between MCF-7 and HUVEC. As a result, we identified 2,080 breast-cancer-specific RNA editing site were detected from MCF-7. These results demonstrate the importance of detecting condition-specific RNA editing sites.

We measured execution times of Expedit and RCARE using a 37.3 Mb sample bam file. Conversion processing of RCARE was performed in 192 seconds on a 3.0 GHz CPU, 2048 MB RAM desktop environment for converting BAM to VCF. RCARE RNA editing site annotations took 7 seconds under 14.19 MBps network environments.

## Discussion

Efficient and user-friendly web-based system for trustworthy RNA-editing-site annotation, comparison and graph visualization. RCARE contains 321,008 human RNA editing sites with rich biological annotations and useful summary plots, as well as *evidence level*s to indicate the reliability of each RNA editing site. Furthermore, it provides a tool for converting sequence-based alignment files into VCF files using a Python script; converted VCF files can be uploaded to the RCARE server for further analysis. The RCARE web interface can be used to easily annotate and visualize VCF-formatted RNA-Seq data, and to download results into CSV and JPG files.

Identification of novel RNA-editing sites became the focus of RNA-editing research about 2~3 years ago [1,5,11]. In recent years, however, the spotlight has shifted to analyzing reliability of identified RNA-editing sites because research revealed that numerous RNA-editing sites were, in fact, false positives. We anticipate that RCARE's *evidence level *will greatly augment the field of RNA-editing site research. RCARE will help toward the identification of trustworthy RNA editing sites.

## Competing interests

The authors declare that they have no competing interests.

## Authors' contributions

S. Lee conceived and designed the system, and also implemented the database and web interface (C. Park assisted the implementation of the database). J. Joung supported the project by providing biological information on RNA editing. S. Lee wrote the paper, with J. Park facilitating the process. J. Kim supervised the project. All authors read and approved the final manuscript

## Supplementary Material

Additional file 1 Number of RNA editing sites for each genomic feature within each *evidence level*. A) Number of RNA editing sites at each *evidence level*. B) *Evidence level *annotations in relation to genomic features. The enrichments for genic features at each *evidence level *from RCARE are also shown. Click here for file

Additional file 2 The ratio of each *evidence level *within three cell line RNA-seq data. A) The ratio of *evidence levels *A-C versus D-E in detected RNA-editing sites within three cell lines including MCF-7 (a breast cancer cell line), HUVEC (a human umbilical vein endothelial cell line) and HeLa-S3 (a cervical carcinoma cell line). B) Number of RNA editing sites at each *evidence level *within three cell llines. Click here for file

Additional file 3 **RCARE webpage user manuals**. Click here for file

Additional file 4 **RCARE convert utilities manuals**. Click here for file

## Declaration

Publication of this article has been funded by the ICT R&D program of MSIP/IITP [B0101-15-247, Development of open ICT healing platform using personal health data].

This research was supported by the ICT R&D program of MSIP/IITP [B0101-15-247, Development of open ICT healing platform using personal health data] and by a grant of the Korean Health Technology R&D Project, Ministry of Health and Welfare (HI13C2164).

This article has been published as part of *BMC Medical Genomics *Volume 8 Supplement 2, 2015: Selected articles from the 4th Translational Bioinformatics Conference and the 8th International Conference on Systems Biology (TBC/ISB 2014). The full contents of the supplement are available online at http://www.biomedcentral.com/bmcmedgenomics/supplements/8/S2.
