# Supplementary figures and images for: RCARE: RNA Sequence Comparison and Annotation for RNA Editing
Source: BMC Med Genomics. 2015 May 29;8(Suppl 2):S8. doi: 10.1186/1755-8794-8-S2-S8 (PMC4460956; doi:10.1186/1755-8794-8-S2-S8)

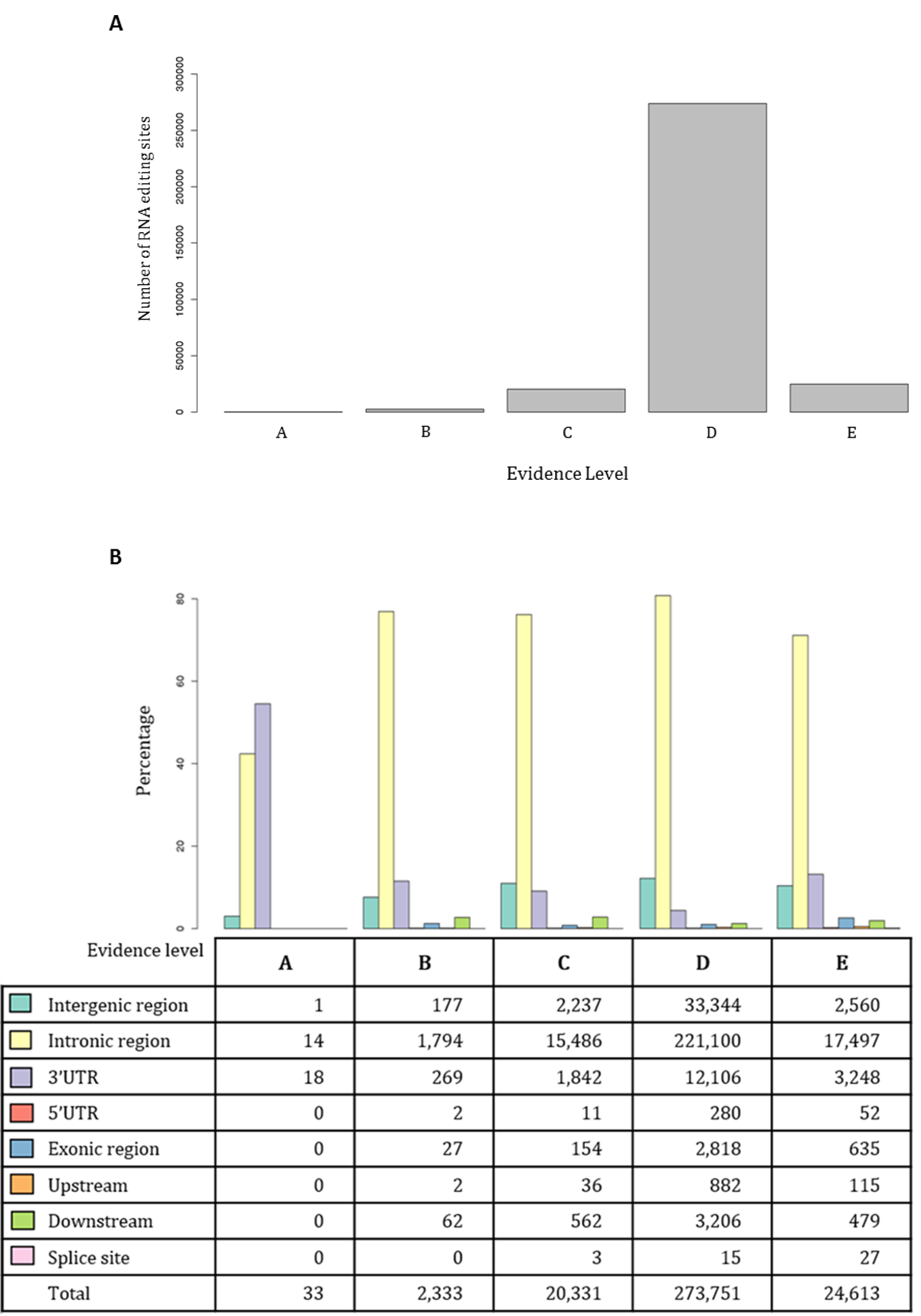

Supplement: Additional file 1 — Number of RNA editing sites for each genomic feature within each evidence level. A) Number of RNA editing sites at each evidence level. B) Evidence level annotations in relation to genomic features. The enrichments for genic features at each evidence level from RCARE are also shown. [file 1755-8794-8-S2-S8-S1.png]

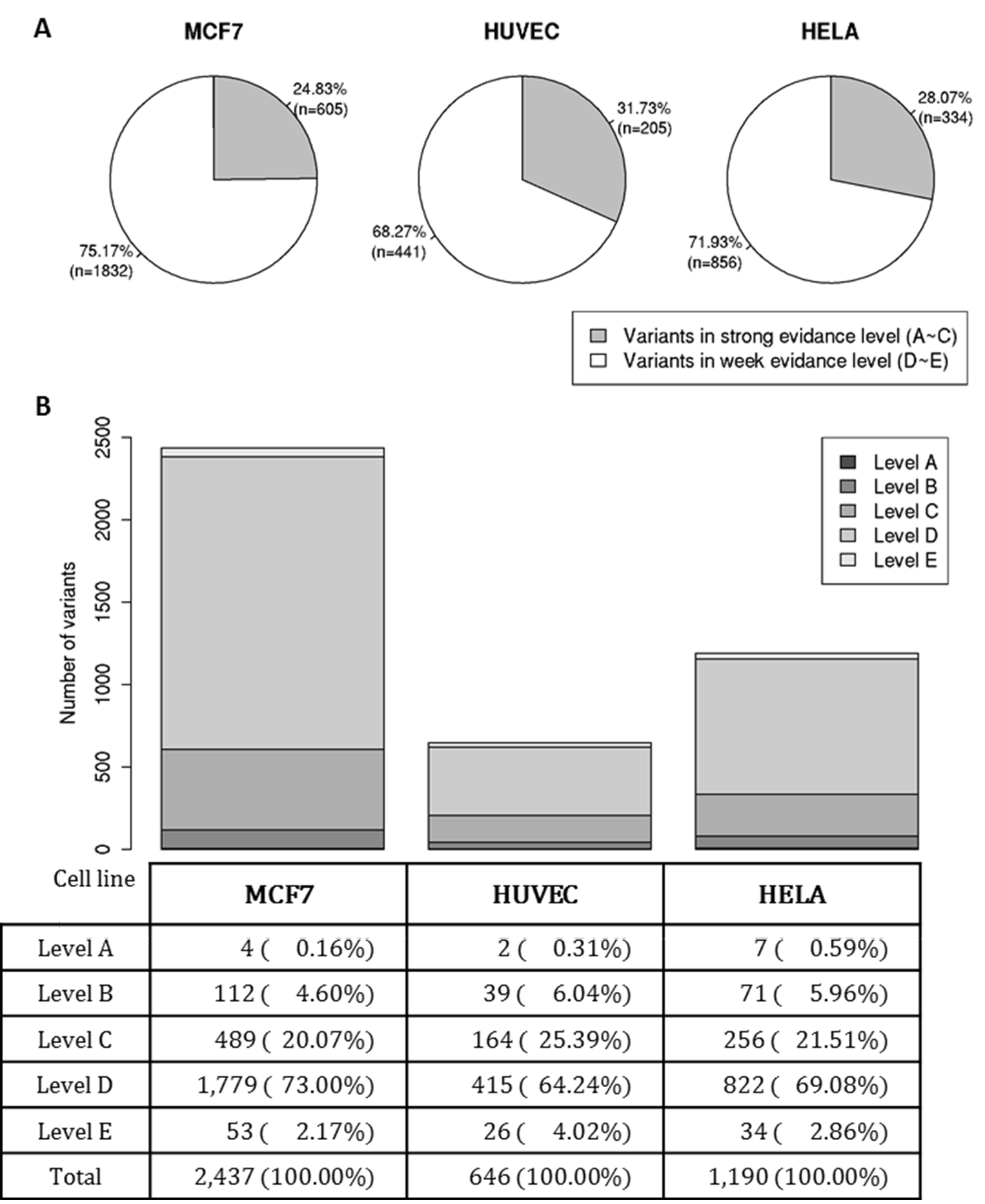

Supplement: Additional file 2 — The ratio of each evidence level within three cell line RNA-seq data. A) The ratio of evidence levels A-C versus D-E in detected RNA-editing sites within three cell lines including MCF-7 (a breast cancer cell line), HUVEC (a human umbilical vein endothelial cell line) and HeLa-S3 (a cervical carcinoma cell line). B) Number of RNA editing sites at each evidence level within three cell llines. [file 1755-8794-8-S2-S8-S2.png]
