# Supplementary material for: RCARE: RNA Sequence Comparison and Annotation for RNA Editing
Source: BMC Med Genomics. 2015 May 29;8(Suppl 2):S8. doi: 10.1186/1755-8794-8-S2-S8 (PMC4460956; doi:10.1186/1755-8794-8-S2-S8)

**Additional File 3. RCARE Webpage user manuals.**

**1. Annotation section**

1. If you have FASTQ or BAM files only, please download the conversion utilities.


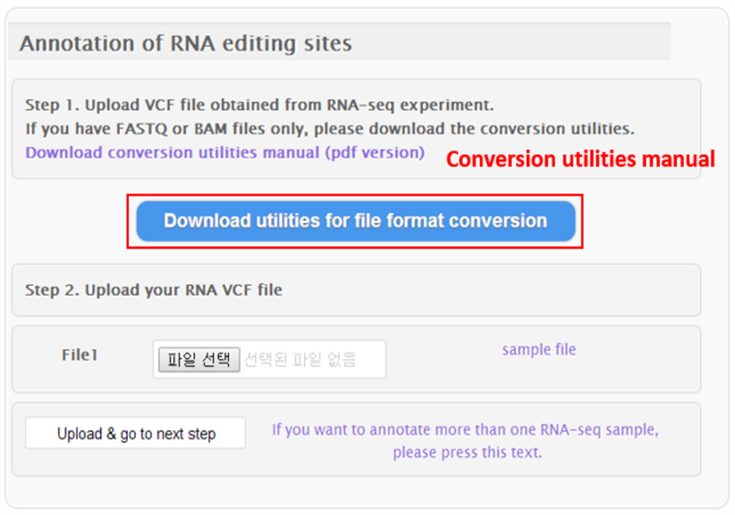


1. Upload a VCF file for RNA editing site annotations.


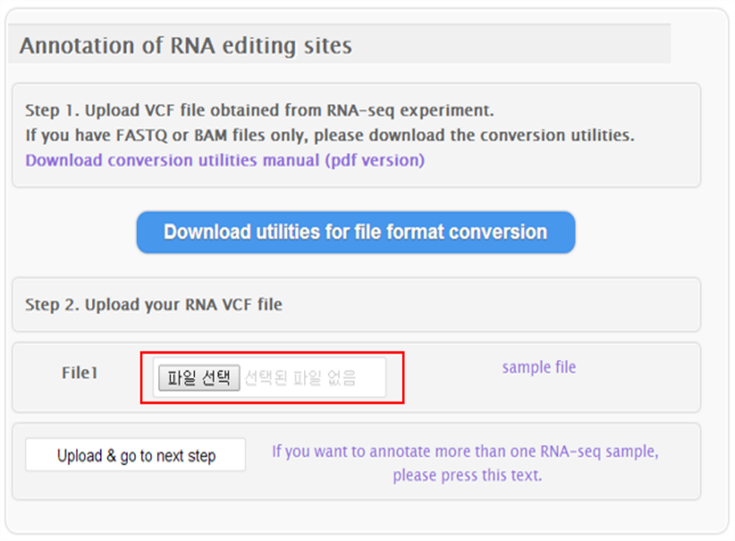


1. Press ‘Upload & to go next step’ button for RNA editing site annotations.


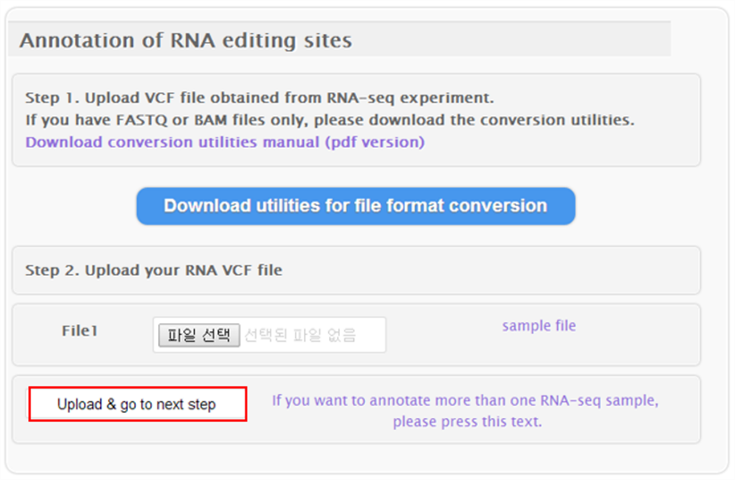


1. Result graphs show annotated results.


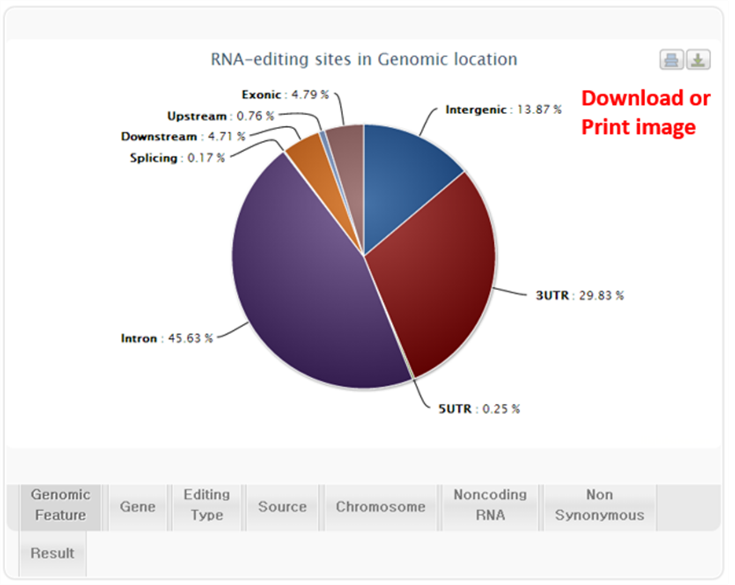


1. Press “*Download result file”* button to download result file.


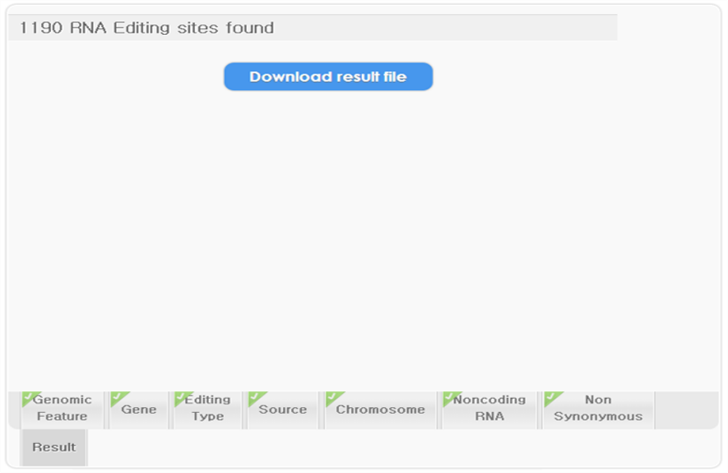


**2. Compare section**

1. If you have FASTQ or BAM files only, please download the conversion utilities.


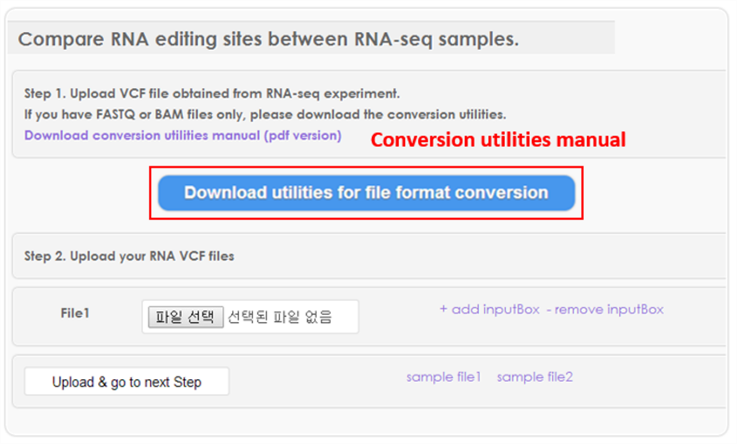


1. Upload a VCF file for annotation of RNA editing sites.


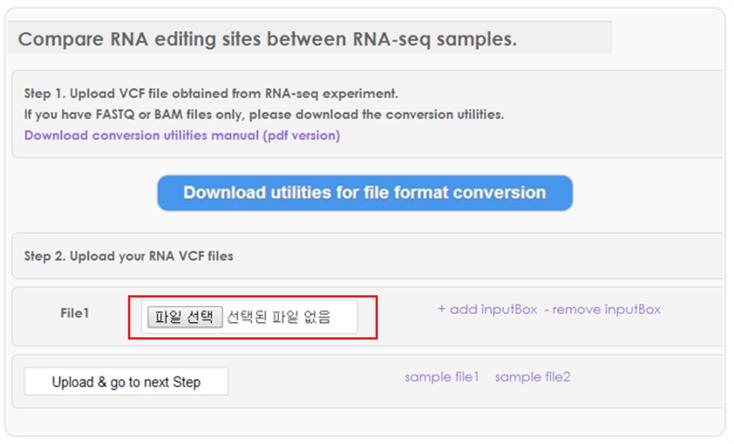


1. Add one more VCF file for comparison.


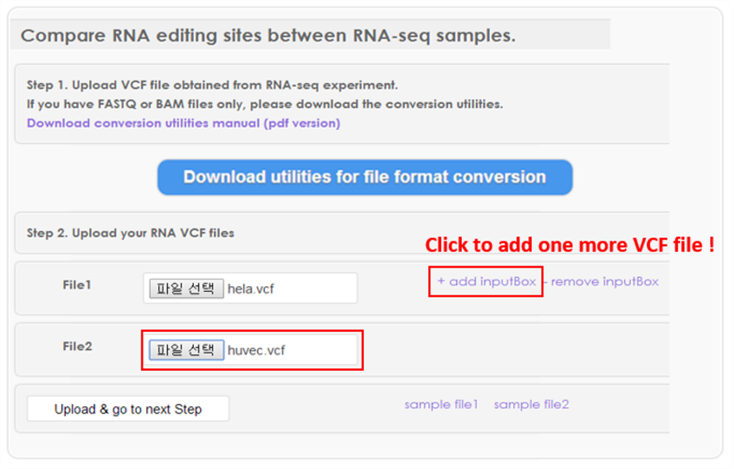


1. Press ‘Upload & to go next step’ button to annotate RNA editing sites.


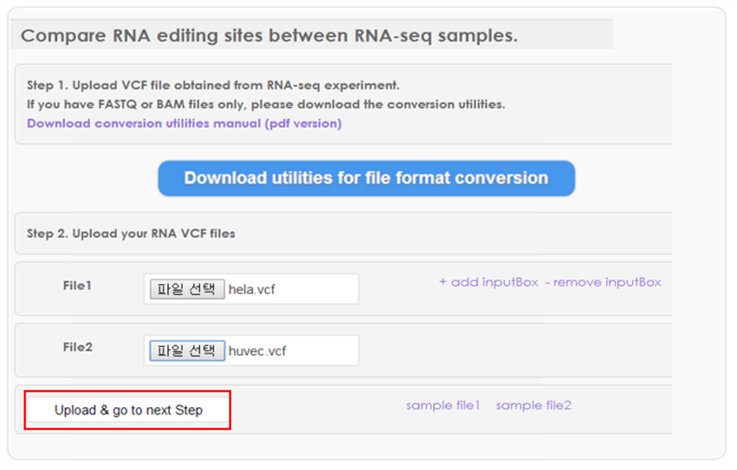


1. Select result files by dragging the files from the right column to the left, and press “*Submit Form”* button.


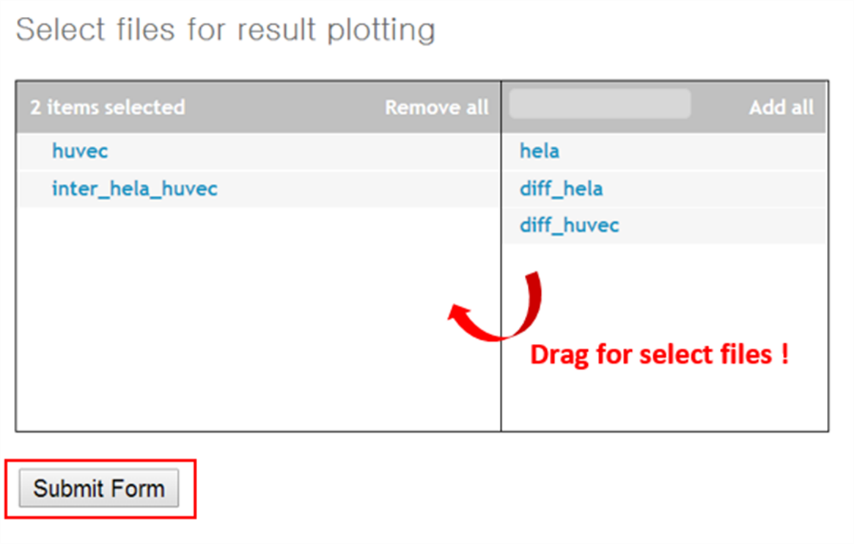


1. Select result graph to view and download result files.


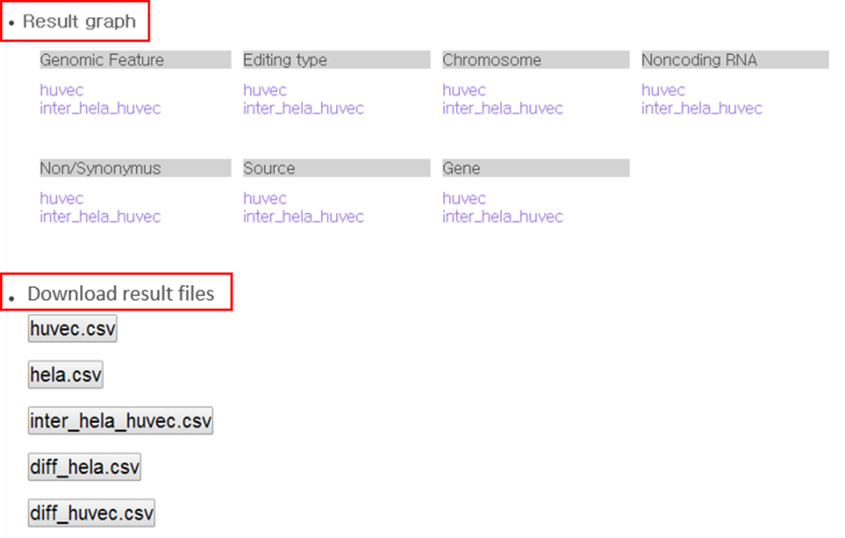


1. Result graphs show annotated results.


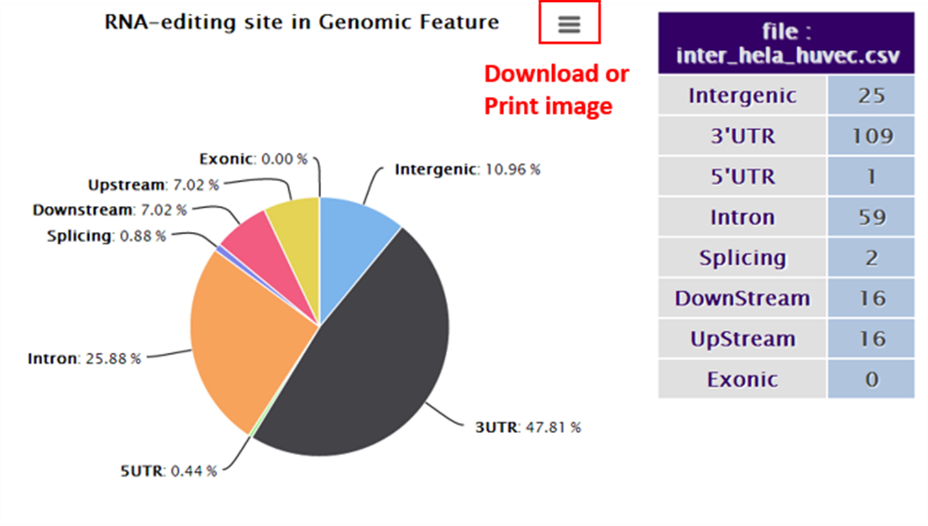

Supplement: Additional file 3 — RCARE webpage user manuals. [file 1755-8794-8-S2-S8-S3.docx]
