# Supplementary material for: RCARE: RNA Sequence Comparison and Annotation for RNA Editing
Source: BMC Med Genomics. 2015 May 29;8(Suppl 2):S8. doi: 10.1186/1755-8794-8-S2-S8 (PMC4460956; doi:10.1186/1755-8794-8-S2-S8)
Supplement: Additional file 4 — RCARE convert utilities manuals. [file 1755-8794-8-S2-S8-S4.docx]

**Additional File 4. RCARE Convert utilities manual.**

**1. Description**

RCARE convert utilities is a set of Python-based utilities for converting FASTQ and BAM (binary format for storing sequence data) into VCF (variant call format) and comparing RNA and DNA VCF files from the same sample. The package provides customized TopHat and SAMtools commands that the user can execute. RCARE convert utilities provides an autoinstallation function for the tools. This is very easy for researchers to use, even for those with no experience of RNA-Seq data analysis.

RCARE convert utilities contains TopHat (http://tophat.cbcb.umd.edu/), SAMtools (http://samtools.sourceforge.net/), Tabix (http://samtools.sourceforge.net/tabix.shtml), VCFtools (http://vcftools.sourceforge.net/), and Bowtie2 (http://bowtie-bio.sourceforge.net/bowtie2). If user presetup tools including TopHat, download light RCARE convert utilities and installation.

**2. Input data format**

RCARE convert utilities convert three sequence formats (FASTQ, BAM, and VCF) to VCF, which is the input format on the RCARE website.

**-** FASTQ format

- FASTQ format is a text-based format for storing both a biological sequence (usually a nucleotide sequence) and its corresponding quality scores.

**-** BAM format

- BAM format is a binary format for storing sequence data.

(http://samtools.sourceforge.net/SAMv1.pdf).

**-** VCF format (variant call format)

- VCF is a text file format (most likely stored in a compressed manner). It contains meta information lines, a header line, and data lines, each containing information about a position in the genome.

**3. Installing and testing the installation**

3-1 Install quick start

- RCARE needs a presetup in the Python environment.

- Download RCARE convert utilities (4.75G) from the website.

- Unzip RCARE convert utilities.

- Tar -xvf RCARE-pre-processing.tar.gz

- Run rcare.py for your purposes.

3-2 Test the installation

The sample BAM data contained only 21 chromosome. These data were extracted from paired-end RNA-Seq using HeLa cells in ENCODE ([http: //genome.ucsc.edu/ENCODE](http://genome.ucsc.edu/ENCODE)).

- Input data confirmation

- ls ./input_data/bam/

- Test command

- python rcare.py -ib sample.bam -fn sample_bam_test

- Result confirmation

- ls ./result_data/vcf/sample_bam_test/

**4. Synopsis and example**

- 1. Input data folder consists of FASTQ, BAM, and VCF. Insert into row data in each folder.
  2. Convert paired-end FASTQ files into VCF format
- Python rcare.py -if -p S1.fastq S2.fastq -fn fastq_test

- Result confirmation

- ls ./input_data/vcf/fastq_test/
  1. Convert single FASTQ file into VCF format
- python rcare.py -if -s S1.fastq -fn single_fastq_test

- Result confirmation

- ls ./input_data/vcf/fastq_test
  1. Convert BAM into convert to VCF format
- python rcare.py -ib sample.bam -fn sample_bam_test

- Result confirmation

- ./result_data/vcf/sample_bam_test

- Compare RNA VCF with DNA VCF file

- python rcare.py -c DNA.vcf RNA.vcf -fn 1_compare_test

- Result confirmation

- ls ./result_data/compare/1_compare_test/
  1. Customized TopHat command running
- python rcare.py -tc "tophat" -fn test
  1. Customized SAMtools command running
- python rcare.py -sc "samtools" -fn test\

**5. RCARE convert utilities options**

| **Option** | **Description** |
| --- | --- |
| -if | Input file format: FASTQ file |
| -ib | Input file format: BAM file |
| -p | Paired end FASTQ file |
| -c | Compare VCF(RNA) with VCF(DNA) |
| -fn | Result file name |
| -tc | Customized TopHat commands |
| -sc | Customized SAMtools commands |

**6. Package composition**

| **Folder file name** | **Description** |
| --- | --- |
| input_data | Insert input data |
| result_data | Save result data |
| resource | Files required for preprocessing |
| tools | Tools required for preprocessing |
| rcare.py | Batch file of convert utilities |

**7. Light RCARE convert utilities installation**

- Download light-RCARE-pre-processing.tar.gz (35.62 MB) from RCARE website.

- Download tools:

1. TopHat: <http://tophat.cbcb.umd.edu/>
2. SAMtools: <http://samtools.sourceforge.net/>
3. Bowtie2: <http://bowtie-bio.sourceforge.net/bowtie2/index.shtml>
4. Tabix: <http://samtools.sourceforge.net/tabix.shtml>
5. VCFtools: <http://vcftools.sourceforge.net/>

- All tools insert into the tools folder in the RCARE convert utilities

- If user has used previous setup tools, initialize each tool’s environment settings

**8. Authors**

Ju Han Kim and Soo Youn Lee from SNUBI (Seoul National University Biomedical Informatics; <http://www.snubi.org/>)

**9. References**

1. Li H, Handsaker B, Wysoker A, Fennell T, Ruan J, Homer N, Marth G, Abecasis G, Durbin R; 1000 Genome Project Data Processing Subgroup. The Sequence Alignment/Map format and SAMtools. Bioinformatics 2009; 25: 2078–9.

2. Trapnell C, Pachter L, Salzberg SL. TopHat: discovering splice junctions with RNA-Seq. Bioinformatics 2009; 25: 1105–11.

3. Danecek P, Auton A, Abecasis G, Albers CA, Banks E, DePristo MA, Handsaker RE, Lunter G, Marth GT, Sherry ST, McVean G, Durbin R; 1000 Genomes Project Analysis Group. The variant call format and VCFtools. Bioinformatics 2011; 27: 2156–8.

4. Langmead B, Salzberg SL. Fast gapped-read alignment with Bowtie 2. Nature methods 2012; 9: 357–9.

5. Li H. Tabix: fast retrieval of sequence features from generic TAB-delimited files. Bioinformatics 2011; 27: 718–9.
